# Indigenous Wild Edible Mushrooms: Unveiling the Chemical Compositions and Health Impacts

**DOI:** 10.3390/foods14132331

**Published:** 2025-06-30

**Authors:** Nattaya Konsue, Sunantha Ketnawa, Si Qin

**Affiliations:** 1 Food Science and Technology Program, School of Agro-Industry, Mae Fah Luang University, Muang, Chiang Rai 57100, Thailand; sunantha.ketnawa@gmail.com; 2 Research Center of Innovative Food Packaging and Biomaterials, Mae Fah Luang University, Muang, Chiang Rai 57100, Thailand; 3 College of Food Science and Technology, Hunan Agricultural University, Changsha 410128, China

**Keywords:** edible wild mushroom, digestive enzyme inhibition, antioxidant activity, cytotoxicity, hepatotoxicity, NF-E2-related factor 2 (Nrf2), quinone oxidoreductase 1 (NQO1)

## Abstract

Wild edible mushrooms (WEMs) are a popular delicacy in Thailand, prized for their unique flavor, texture, and nutritional value. Despite their widespread consumption, there is limited scientific research on their chemical compositions, biological activities, and potential health benefits. To bridge this knowledge gap, a comprehensive study was conducted on sixteen WEM species from ten families—Polyporaceae, Pleurotaceae, Russulaceae, Marasmiaceae, Pluteaceae, Boletinellaceae, Diplocystaceae, Lyophyllaceae, Psathyrellaceae, and Auriculariaceae—commonly found in northern Thailand. The proximate composition varied significantly among the WEM species, particularly in crude protein (12–51% *w*/*w*), crude fiber (1–30% *w*/*w*), and glucans (4–25% *w*/*w*). *Astraeus odoratus* exhibited the highest phenolic content, while *P.* cf. *portentosus* demonstrated the most potent antioxidant activity. WEM extracts also displayed notable inhibitory effects on α-glucosidase (5.82–79.43%) and α-amylase (1.30–90.79%). All extracts induced antioxidant regulators of Nrf2 and NQO1, suggesting that WEMs can help protect cells from oxidative stress, environmental toxins, and xenobiotics from food. Importantly, all extracts maintained high cell viability (>80%), indicating their safety for consumption. Furthermore, the mushrooms demonstrated a strong ability to reduce hepatotoxicity in HepG2 cells induced by tert-butyl hydrogen peroxide, highlighting their potential in preventing liver damage. This study not only underscores the nutritional and health benefits of WEMs but also establishes a vital scientific foundation for future research on their health effects and in vivo applications. In turn, these findings could serve as a crucial resource for optimizing the use of WEMs in ethnic cuisines and strengthening claims regarding their functional food properties.

## 1. Introduction

A wild edible mushroom (WEM) refers to any type of mushroom that is collected from natural deposits [1]. Indigenous communities have traditionally utilized wild edible mushrooms as a supplementary food source alongside plant- and animal-derived foods [2]. A diverse range of forest types flourish in northern Thailand, characterized by varying amounts of wetness and magnitudes of disturbance. Although culinary wild mushrooms are considerably more expensive than cultivated mushrooms, they continue to be popular among customers because of their unique characteristics, taste and texture. Genera such as *Russula* and *Lactarius*, known for their bitter flavor, have inspired the development of local cuisine, resulting in flavorful dishes. Meanwhile, the diversity of wild edible mushrooms in Asia is high, with specific varieties boasting notable gastronomic or medicinal values. Surprisingly, the percentage of novel species is generally as high, ranging from 55% to 96% [3]. Furthermore, a separate study in the Chiang Mai community forest area identified 58 previously unrecognized wild mushroom species in the region [4]. Despite these findings, the process of taxonomy remains a bottleneck, hindering the formal recognition and classification of new species. However, it is more important to focus on understanding how to safely consume these species and to investigate their chemical compositions and health impacts, providing consumers with essential information.

Currently, global demand for mushrooms is steadily increasing, driven by their nutritional value, health benefits, and diverse applications. Mushrooms are marketed in various forms—including fresh, dried, dietary supplements, and extracts—and are consumed worldwide [5]. Nutritionally, they are low in fat but rich in dietary fiber, essential vitamins (B1, B2, B12, C, D, and E), and minerals such as potassium, iron, copper, zinc, and manganese [6,7,8]. In addition, mushrooms provide high-quality protein containing all essential amino acids, making them a valuable food source for addressing food security and promoting sustainable diets, particularly in resource-limited settings [9].

What distinguishes mushrooms from other functional foods is their rich diversity of secondary metabolites, which contribute significantly to their pharmacological potential. Beyond their culinary appeal, mushrooms are increasingly recognized for their nutraceutical properties and bioactive compound content. Researchers have identified a range of bioactive constituents including high- and low-molecular-weight polysaccharides, protein-bound polysaccharides, glycoproteins, triterpenoids, and immunomodulatory proteins [10,11]. Among these, compounds such as β-glucans, enzymes, and various secondary metabolites play a central role in their symbiotic and health-enhancing properties [12,13,14,15]. Of particular interest are β-glucans, triterpenoids, and phenolic compounds, which have been widely studied for their functional bioactivities. β-Glucans modulate immune responses and promote apoptosis, contributing to anti-cancer and immune-enhancing effects [16]. Triterpenoids, especially abundant in wild *Ganoderma lucidum*, exhibit potent anti-inflammatory and lipid-regulating activities by influencing NF-κB signaling and metabolic enzyme pathways [17]. Phenolic compounds, such as gallic acid and catechins, contribute significantly to antioxidant activity through free radical scavenging [18]. These bioactive compounds may also function synergistically, enhancing therapeutic efficacy. For example, in *Pleurotus ostreatus*, the combination of polysaccharides and phenolics has been shown to improve glycemic regulation and oxidative balance [18]. As a result, WEMs have attracted attention not only as functional food ingredients but also as promising sources of natural therapeutics and nutraceuticals.

Despite the widespread consumption of WEMs in Thailand, there is a notable lack of scientific evidence regarding their chemical compositions, biological activities, and potential health benefits. Although these mushrooms are widely believed to be rich in nutritional value and contain a diverse range of bioactive compounds that could positively impact human health, there is still a significant gap in tangible proof and a comprehensive database. Given their potential, it is crucial to fill this gap with rigorous scientific investigation. Therefore, this study focuses on 16 wild mushroom species collected from the northern region of Thailand, selected based on their potential health benefits. The research aims to thoroughly investigate their morphological characteristics, chemical compositions, and health-promoting properties, with particular attention to bioactive compounds such as polysaccharides and antioxidants. This research is vital to validate the health claims surrounding these mushrooms and to build a strong scientific foundation for their use in both local and global contexts.

## 2. Materials and Methods

### 2.1. Chemical Reagents

Minimum essential medium (MEM) and fetal bovine serum (FBS) were obtained from Gibco (Waltham, MA, USA). Tert-butyl hydrogen peroxide (tBPH), penicillin/streptomycin, 0.25% trypsin, and the CCK8 kit were purchased from Sinopharm (Wuhan, China). α-Amylase from *Bacillus* sp. (≥400 units/mg protein), α-glucosidase from *Saccharomyces cerevisiae* (≥10 units/mg protein), and acarbose were purchased from Sigma-Aldrich (St. Louis, MO, USA). 2.2-Diphenyl-1-picrylhydrazyl (DPPH), tripyridyl triazine (TPTZ), and 6-hydroxy-2,5,7,8-tetramethylchroman-2-carboxylic acid (Trolox), ethyl alcohol, quercetin, gallic acid, Griess reagent, and lipopolysaccharide were procured from Sigma-Aldrich (Buchs, Switzerland). Other chemicals were all analytical grades. . 

### 2.2. Collection and Preparation of Sample

Sixteen WEM species from ten families—Polyporaceae, Pleurotaceae, Russulaceae, Marasmiaceae, Pluteaceae, Boletinellaceae, Diplocystaceae, Lyophyllaceae, Psathyrellaceae, and Auriculariaceae—were obtained from local markets in the north of Thailand from June to July 2023 in the rainy season. After being collected, mushroom samples were promptly brought to the laboratory and processed within a short period. Every fruiting body underwent a meticulous cleansing process before drying at a temperature of 50 °C using a tray dryer (Kluay Nam Thai Trading Group Co., Ltd., Bangkok, Thailand) for 24 h. The dried fruiting bodies were subsequently pulverized to fine powder as “crude sample” and used for proximate analysis. One hundred grams of the crude sample powder was extracted with 1 L of 100 °C water for 4 h. The samples were filtered and freeze-dried to obtain the “lyophilized sample”. For bioactivity assays, the lyophilized extract was further extracted with methanol (lyophilized sample + methanol). All sample extracts were stored in a freezer at −20 °C until further analysis.

### 2.3. Morphological and Molecular Identification

Morphological identification involves taxonomic identification using a combination of micro and macro morphology. Fresh mushrooms were identified based on the macroscopic and/or microscopic characteristics of their fruiting bodies. The taxonomic literature utilized for morphological species identification was referenced from the book “How to Identify Mushrooms to Genus” [19].

Molecular identification involved DNA extraction from each sample, which was conducted using the highly pure PCR template preparation kit (Hoffmann-La Roche Ltd., Basel, Switzerland), following the manufacturer’s protocol. Subsequently, the DNA extracted from each sample was amplified utilizing primers specific for ribosomal DNA regions (ITS1/ITS4) [20]. The resulting PCR products were tested by SolGent Co., Ltd., Daejeon, Republic of Korea, for sequencing. The identification process followed the method described by Hall [21], which involves combining the sequencing data and conducting a Blast search to find the closest matches [21].

### 2.4. Analysis of Proximate Compositions

Three grams of each crude sample was analyzed for their proximate contents according to the Association of Official Analytical Chemists (AOAC) method [22]. The moisture content was determined by hot air oven drying at 110 °C for 1 h, while crude fiber was analyzed using dilute acid and alkali hydrolysis. The crude fat content was conducted through the acid hydrolysis method, whereas the micro Kjeldahl method was employed to obtain crude protein (%Protein = N × 4.38), and the ash content was carried out by ashing at 550–600 °C for 3 h. The result was calculated based on a dry-weight basis.

### 2.5. Analysis of Glucan

The β-Glucan Assay Kit (Yeast and Mushroom) (K-YBGL, Megazyme Ltd., Wicklow, Ireland) was employed to measure the glucan content in lyophilized samples. The assays were conducted following the instructions provided by the manufacturer. Subsequently, the absorbance of the resulting color complex was determined at 510 nm using a microplate reader. The concentration of total glucan (*w*/*w*) and α-glucan (*w*/*w*) was directly determined, while the β-glucan content (*w*/*w*) was approximated by subtracting the α-glucan content from the total glucan content. These results were expressed as a percentage of dry weight (DW).

### 2.6. Analysis of Total Phenolic Content (TPC)

Prior to analysis, 30 mL of lyophilized extract was mixed with 1.5 mL of 80% methanol. Samples were then homogenized using a mixer for 5 min. The extraction was further facilitated by subjecting samples to ultrasonic bath treatment for 15 min and then mixing in a rotation homogenizer for 1 h at 150 rpm. Subsequently, samples were centrifuged at 3000× *g* for 10 min (5424-R, Eppendorf Co., Ltd., Hamburg, Germany), and the supernatants were collected for further analysis.

The total polyphenolic content (TPC) was determined using spectrophotometry, employing gallic acid as the reference standard following the method according to Aumasa, Ogawa [23]. Briefly, 0.5 mL of the lyophilized extract (20 mg/mL) was transferred to a tube containing 2.5 mL of a diluted solution of Folin–Ciocalteu’s reagent in distilled water. Subsequently, 2.0 mL of a sodium carbonate solution (7.5% *w*/*v*) was added. The tubes were then allowed to stand at room temperature for 60 min. Sample extracts of 250 μL were transferred to a 96-well plate, and the absorbance was measured at 765 nm using a microplate reader (Multiskan GO, Thermo Fisher Scientific Inc., Vantaa, Finland), with distilled water as the blank. The TPC was expressed as mg gallic acid equivalents (GAE) per gram of sample.

### 2.7. Determination of Phenolic Profile by Liquid Chromatography–Mass Spectrometry (LC-MS)

The phenolic compound analysis was conducted employing liquid chromatography–mass spectrometry (LC-MS) using an LCMS-8060 instrument by Shimadzu, Kyoto, Japan. One milligram of lyophilized extract was dissolved in 1 mL of 80% methanol and then filtered through a 25 mm syringe filter and transferred into a vial. One microliter of each sample was injected through an autosampler system. Separation was carried out on an ACE Excel C18 column (2.1× *g* 100 mm, particle size 1.7 μm) maintained at 30 °C. The mobile phases consisted of 0.1% formic acid in distilled water (*v*/*v*) (A) and acetonitrile (B). A binary gradient program was employed, initiating with 10% B at 0.30 min. The percentage of mobile phase B was subsequently increased to 15% at 2.40 min, 20% at 3.25 min, maintained at 20% until 3.60 min, raised to 95% at 6.20 min, and maintained at 95% until 7.00 min. At 7.50 min, the initial conditions were restored by decreasing the percentage of mobile phase B from 95% to 10%, followed by column equilibration until 11.00 min. The flow rate was consistently set at 0.3 mL/min for all separations. Electrospray ionization (ESI) in negative mode was employed for MS fragmentation, with the acquisition mode set to multiple-reaction monitoring (MRM) and an interface voltage of 4000 V. Absorbance spectra were collected within the 200–600 nm interval. The peak area of each compound was compared to the calibration curve obtained from the external standard at concentrations ranging from 0.01 to 5 µg/mL. The concentration was calculated based on the linear relationship (y = mx + c) between the peak area and concentration. Identified phenolic compounds were then expressed as mg/L. The limit of detection (LOD), retention time, and chromatogram of each detectable phenolic compound are shown in Table S1 and Figure S1 in the Supplementary Material.

### 2.8. Analysis of Antioxidant Activities

The DPPH radical scavenging activity assay was carried out by the method according to Aumasa, Ogawa [23]. First, 50 μL of the lyophilized extract (20 mg/mL) was mixed with 1950 μL of freshly prepared DPPH 60 mM solution, which was prepared by dissolving 2.36 mg of DPPH in methanol to a total volume of 100 mL. The mixture was then incubated at room temperature in a dark place for 30 min. The absorbance was measured at a wavelength of 517 nm using a microplate reader. A sample blank was prepared using 95% methanol instead of the DPPH solution, following the same procedure. A standard curve was prepared using Trolox in the range of 200–1000 μM. The activity was calculated after subtracting the sample blank and expressed as mmol Trolox equivalents (TE) per g of sample.

The ferric-reducing antioxidant power assay (FRAP) was performed according to Aumasa, Ogawa [23]. The procedure involved preparing the FRAP reagent by mixing 300 mM acetate buffer (pH 3.6), 40 mM HCl, 10 mM 2,4,6-tripyridyl-s-triazine (TPTZ), and 20 mM FeCl_3_ at a ratio of 10:1:1. Subsequently, 400 μL of the lyophilized extract was mixed with 2.6 mL of the FRAP reagent solution, vortexed for 60 s, and then incubated at 37 °C for 30 min. The absorbance against a blank was measured using a UV-spectrometer (Thermo Fisher Scientific Ltd., Ratastie, Finland) at a wavelength of 595 nm. The FRAP value was calculated as mmol of ferrous sulfate (FeSO_4_) per g of sample.

### 2.9. Analysis of Key Antioxidant Regulators by Western Blot

The analysis of key antioxidant regulators by western blot followed the method described by Yi, Li [24]. Protein extraction from lyophilized samples was carried out using the total protein extraction kit (Sola Biosciences, Beijing, China) as per the manufacturer’s instructions. The cells were lysed on ice with Radioimmunoprecipitation assay (RIPA) buffer (Beyotime, Shanghai, China), and the protein concentration was measured using a bicinchoninic acid (BCA) assay kit (Biosharp, Shanghai, China). The extracted proteins were then subjected to sodium dodecyl sulfate–polyacrylamide gel electrophoresis (SDS-PAGE) with 15% gels, followed by transfer onto polyvinylidene difluoride (PVDF) membranes (Amersham Pharmacia Biotech, Little Chalfont, UK). The membranes were blocked in skim milk for 1 h at room temperature and then incubated overnight at 4 °C with primary antibodies. Antibodies against Nrf2 and NQO1 were sourced from Cell Signaling Technology (Beverly, MA, USA) and used at a 1:1000 dilution, while the β-actin antibody from Proteintech (Wuhan, China) was used at a 1:20,000 dilution. After washing with Tris-buffered saline with Tween 20 (TBST), the membranes were incubated with the appropriate secondary antibody for 1 h at room temperature. Images were captured using an Image Quant LAS 4000 mini (General Electric Medical System Co., Ltd., MA, USA) and visualized with an ECL reagent (Xin-Sai-Mei, Suzhou, China).

### 2.10. Digestive Enzyme Inhibition Assays

The α-amylase inhibitory activity assay was conducted following the methods of Johnson, Lucius [25]. Briefly, 150 μL of lyophilized sample dissolved in distilled water at a concentration of 0.063 mg/mL was combined with 50 μL of enzyme solution (α-amylase) at a concentration of 2 U/mL. Acarbose (0–10 mg/mL) was employed as the positive control against α-amylase. The mixture was incubated at 37 °C for 10 min. Then, 50 µL of a 0.05% starch solution was added and incubated at 37 °C for 10 min. The reaction was finalized by the addition of 100 µL of dinitrosalicylic acid solution. The absorbance at 565 nm was measured. The IC_50_ value was determined in triplicate, and the percentage inhibition of enzyme activity was calculated using the equation below.

α-Amylase inhibitory activity (%) = [(A_control_ − A_sample_)/A_control_] × 100

The α-glucosidase inhibitory activity assay was also conducted according to the method of Johnson, Lucius [25]. Briefly, 50 μL of lyophilized extract (0, 0.2, 0.4, 0.6, 0.8, 1.0 mg/mL) was mixed with 100 μL (1.0 U/mL) of enzyme solution (α-glucosidase). Acarbose (0–10 mg/mL) was employed as the positive control against α-glucosidase. The mixture was incubated for 10 min. Then, 50 μL of a 5 mM p-nitrophenyl-α-d-glucopyranoside solution was added and incubated at 25 °C for 5 min. The reaction was terminated by the addition of 2 mL of 200 mM Na_2_CO_3_ prior to measuring the absorbance at 405 nm. The IC_50_ value was determined in triplicate, and the percentage inhibition of enzyme activity was calculated following the equation below.

α-Glucosidase inhibitory activity (%) = [(A_control_ − A_sample_)/A_control_] × 100

### 2.11. Cytotoxicity and Hepatotoxicity Prevention Effect

The cytotoxicity of lyophilized samples dissolved in distilled water at concentrations of 0–1 mg/mL on HepG2 cells was determined using the method described by Mir, Elieh Ali Komi [26]. HepG2 cells were obtained from the Cell Bank of Type Culture Collection of the Chinese Academy of Sciences (Shanghai, China). These cells were cultured in MEM supplemented with 10% FBS and 1% penicillin/streptomycin within a humidified atmosphere containing 5% CO_2_ at 37 °C. Upon reaching approximately 90% confluency, the cells were removed using 0.25% trypsin and subsequently grown in cell culture plates.

Briefly, cells were seeded in 96-well plates at a density of 4 × 10^3^ cells per well and allowed to grow for 24 h. The treatment groups were pre-incubated with or without 0.02–1.00 mg/mL lyophilized extracts for an additional 24 h. Afterward, the medium was removed, and the cells were co-incubated with 110 μL of fresh medium containing 10 μL of CCK8 for 1 h. Finally, the absorbance was measured at 450 nm with a microplate reader. Each treatment was performed in triplicate.

The hepatotoxicity protection effects of lyophilized samples dissolved in distilled water at a concentration of 0.02 mg/mL against tBPH were measured by the CCK8 assay according to Wu, Yong-Jun [27]. Briefly, cells were grown in 96-well plates at a density of 4 × 10^3^ cells per well for 24 h. The treatment group was pre-incubated with lyophilized extract for an additional 24 h prior to exposure to 175 μM tBPH for 3 h. The model group was treated solely with tBPH for 3 h, while catechin (10 µmol/L) served as a positive control. The subsequent steps followed the protocol outlined in the section above.

### 2.12. Statistical Analysis

Statistical analysis was carried out using Microsoft Excel 2010 (Microsoft Cooperation, WA, USA) and SPSS 22.0 (IBM Corporation, NY, USA). All extractions and analyses were performed in triplicate, and the data are shown as the mean ± standard deviation (SD). Values not sharing a common letter are significantly different at *p* < 0.05 using one-way ANOVA and post hoc multiple mean comparisons (Tukey’s HSD test).

## 3. Results and Discussion

### 3.1. Morphology and Molecular Identification

The PCR products derived from wild edible mushrooms (WEMs) were identified by analyzing the sequencing data and performing a Blast search to identify the closest matches, as detailed in Table 1. Certain WEMs can be identified based on their morphology due to their distinct characteristics including *Pleurotus ostreatus*, *Pleurotus* sp. 2, *Pleurotus* sp. 3, *Russula* cf. *emetica*, *Phlebopus* cf. *portentosus*, *Termitomyces* cf. *fulizinosus*, and *Coprinopsis cinerea.* The DNA sequences revealed the exact sequence of bases (A, C, G, and T) in a DNA molecule of WEMs, ranging from 387–646 base numbers. In this study, WEMs were categorized in 10 different families and 16 species: Polyporaceae (*L. squarrosulus*, *L. sajor-caju*, *L. polychrous*), Pleurotaceae (*P. ostreatus*, *P.* sp1, *P.* sp2), Russulaceae (*R.* cf. *emetica*, *R.* sp1, *R.* sp2), Marasmiaceae (*L. edodes*), Pluteaceae (*V*. *volvacea)*, Boletinellaceae (*P.* cf. *portentosus*), Diplocystaceae (*A. odoratus*), Lyophyllaceae (*T.* cf. *fulizinosus*), Psathyrellaceae (*C. cinerea*), and Auriculariaceae (*A. cornea*). The abundance of each species is distinct based on various factors. The primary factors influencing wild mushroom production include weather conditions, nutrient availability, forest management practices, and the characteristics of the forest stands, although the ambient temperature, soil moisture, and precipitation are considered to be the most important factors that account for variations in mushroom yields from year to year [28]. Non-mycorrhizal mushrooms including *Volvariella* spp., *Lentinus edodes*, *Pleurotus* spp., *Auricularia* spp., and *Agaricus* spp. are available year-round in the market in northern Thailand [29]. In contrast, most edible wild mushrooms are only abundant during the rainy season, from June to October. Despite commanding higher prices than cultivated mushrooms, wild mushrooms are preferred for their superior palatable flavor and texture.

*Pleurotaceae* is one of the most abundant families. Specifically, *P. ostreatus* is commonly found in local markets in Chiang Mai, Thailand. *Russulaceae* is a large group that includes milk-cap mushrooms, of which over 100 species have been identified, primarily in the four northern provinces of Thailand: Chiang Mai, Chiang Rai, Mae Hong Son, and Lampang, since 2007 [30]. A total of 22 species belonged to the genus *Amanita*, followed by 20 species from the genus *Russula*, which were morphologically identified and combined with the local wisdom data from 15 sites of natural and community forests located in 12 Provinces of Thailand: Chiang Mai, Chiang Rai, Mae Hong Son, Nan, Maha Sarakham, Ubon Rachathani, Yasothon, Loei, Mukdahan, Sakon Nakon, Burirum, and Surat Thani, according to a study by Naksuwankul, Thongbor [31]. Furthermore, Kumla, Suwannarach [3] collected WEMs including a total of 19 specimens of edible *Amanita* in northern Thailand during the period from 2019 to 2022 and reported the morphological characteristics classified as *A. hemibapha*, *A. pseudoprinceps*, *A. rubromarginata*, *A. subhemibapha*, and *Amanita* section *Caesareae*.

### 3.2. Chemical Composition

Table 2 illustrates the macronutrients and functional polysaccharides presented in WEM crude samples. The protein content in WEMs varied significantly among species, ranging from the lowest value of 12.02 ± 0.26 g/100 g DW for *A. cornea* to the highest value of 51.87 ± 1.05 g/100 g DW for *C. cinerea*. Interestingly, slight variations in the protein content were observed even within the same family, such as in the *Russulaceae* (ranging from 30.15 ± 0.39 to 31.74 ± 2.33 g/100 g DW) and *Pleurotaceae* (ranging from 22.10 ± 0.17 to 26.74 ± 0.26 g/100 g DW) families. This is in correlation with the protein contents of 17.06 g/100 g DW observed in *P. ostreatus* in a previous study [32]. The protein content of mushrooms is significantly influenced by factors such as species, feed composition, the harvest time, and environment, typically ranging between 19 and 35 g/100 g DW [33]. Our current investigation suggests that WEMs can serve as a valuable protein source for human health. Hence, they are considered safe sources of high-quality proteins with a low fat content. Currently, edible mushrooms are increasingly being utilized as an alternative source of animal protein. With advancements in non-animal-based meat production, it may soon be possible to create mushroom protein meat analogs that closely mimic the taste and flavor of animal meat. This advancement is anticipated to considerably improve the nutritional value of meals derived from edible mushrooms. Since then, mushroom proteins have garnered favor in the food business owing to their nutritional benefits and comprehensive necessary amino acid profile.

Moreover, each WEM might exhibit a different polysaccharide composition. The experimental results showed that the total glucan content in WEMs varied widely, from 3.99 ± 0.99% *w*/*w* for *A. odoratus* to 30.21 ± 6.01% *w*/*w* for *V. volvacea*. β-Glucan pre-dominated in most WEMs, ranging from 2.21 ± 1.00% *w*/*w* to 23.86 ± 7.66% *w*/*w*. From the previous studies, *L. edodes* has been shown to possess 26.7% β-glucan on a dry weight basis [34], similar to the current result. On the other hand, α-glucan was present in lower amounts in mushrooms, ranging from 0.43 ± 0.09% *w*/*w* to 8.40 ± 1.36% *w*/*w*. This aligns with the findings of Mirończuk-Chodakowska and Witkowska [35], who illustrated the predominance of β-glucan composition in wild edible mushrooms, such as those within the Russulaceae family and *Tricholomopsis rutilans*, which have β-glucan levels up to 40.9% [36]. In addition, commonly consumed *Agaricus bisporus* exhibited lower glucan concentrations, varying from 8.6% to 12.3% depending on the studied portion. This suggests that some wild species may serve as a viable alternative source of glucans for functional food applications, particularly due to their natural abundance and nutritional value. A published paper revealed the carbohydrate composition of *V. volvacea*, which includes principally polysaccharides such as glycogen; hemicelluloses such as mannans, xylans, and galactans; and other indigestible fibers such as chitin, cellulose, glucans, and dietary fibers [37]. Bioactive polysaccharides such as glucans are renowned for their antioxidative and anticancer properties, as well as their capacity to enhance the immune system. These beneficial attributes are derived from their chemical structure, characterized by glucose residues linked via β-(1–3)-glycosidic bonds and sporadic β-(1–6) branching points [38].

Notably, different species of WEMs showed significant variation in the crude fiber content. The crude fiber content of lyophilized extracts from WEMs ranged substantially across species, from the lowest at 0.91 ± 0.05 g/100 g DW for *T.* cf. *fulvinosus* to the highest at 31.14 ± 3.67 g/100 g DW for *A. odoratus*. The top five species with the highest crude fiber content in this study were A. *odoratus*, *A. cornea*, *P. ostreatus*, *L. sajor-caju*, and *P.* sp.3, at 31.14, 28.94, 23.68, 23.42, and 23.26 g/100 g DW, respectively. In particular, within the same family of Polyporaceae, the crude fiber contents of *L. squarrosulus*, *L. sajor-caju*, and *L. polychrous* showed slight differences ranging from 21.26 ± 0.86 to 30.08 ± 1.34 g/100 g DW. Previous studies indicated that the crude fiber content of the fruiting bodies of *L. edodes* ranged from 6.50 to 14.7 g/100 g DW [39], which is lower than the 17.65 g/100 g DW found in our study. In addition, the observed fiber content followed this sequence: *A. odoratus* > *L. polychrous* > *L. squarrosulus* > *L. edodes* [39]. Consequently, *A. adoratus* is considered an excellent source of dietary fiber. The crude fiber content can vary depending on the WEM species. The results support sustainability in food systems, as increasing fiber consumption can enhance public health and improve the functionality and quality of both conventionally processed meat products and plant-based meat alternatives.

Our investigation also revealed varying crude fat contents, ranging from 0.52 ± 0.01 g/100 g DW for *P.* cf. *portentosus* to 4.71 ± 0.03 g/100 g DW for *R.* sp. 3. These findings are consistent with the existing literature. For instance, *P. ostreatus* in our study showed a crude fat content of 1.21%, which is comparable to previous studies, indicating crude fat contents of 1.51% [40], 1.34% [41], and 1.75% [42]. The low crude fat content observed in mushrooms suggests its potential benefits in managing obesity and cardiovascular diseases.

Furthermore, the ash content, indicating the non-combustible residue of a substance, reflects the presence of diverse minerals essential for immune regulation, homeostasis maintenance, disease prevention, and metabolic function [43]. The ash contents in WEMs varied slightly among species, ranging from 2.52 ± 0.04 g/100 g DW for *A. cornea* to 11.49 ± 1.13 g/100 g DW for *A. odoratus*. A previous study demonstrated that *L. squarrosulus* and *V. volvacea* contain significant amounts of phosphorus, potassium, calcium, and sodium, all suitable for daily dietary intake [44]. This suggests their potential as sources of high-quality food, supporting various biological roles beneficial to human health. Nevertheless, the concentration of trace metals in mushrooms is influenced by factors such as the mushroom species, location, the age of the fruiting bodies and mycelium, and the presence of sources of contamination, such as agricultural chemicals and heavy metal-enriched soils [44].

In summary, the contents of macronutrients present in WEMs were found in the following order: crude protein, crude fiber, ash, and crude fat. The results revealed that the protein content was the highest, ranging from 12.02 ± 0.26 to 51.87 ± 1.05 g/100 g DW, followed by the crude fiber (0.91 ± 0.05 to 30.08 ± 1.34 g/100 g DW), ash (2.52 ± 0.04 to 11.49 ± 1.13 g/100 g DW), and crude fat (0.52 ± 0.01 to 4.71 ± 0.03 g/100 g DW). These findings substantiate that WEMs are a rich source of macronutrients, positioning them as a promising candidate for promoting the consumption of WEMs and further applications in the food industry.

### 3.3. Total Phenolic Content and Antioxidant Activity

Hot water extraction was employed to extract constituents from the WEM cell matrices, including polysaccharides, proteins, fats, and others. This method has been shown to potentially enhance the functional properties of mushrooms, such as improving their total polysaccharide content, influencing monosaccharide compositions, and molecular weight, and improving water solubility [45]. Furthermore, our study provides data based on non-solvent extraction, offering insights that are directly applicable to real-world human consumption.

**Figure 1:**
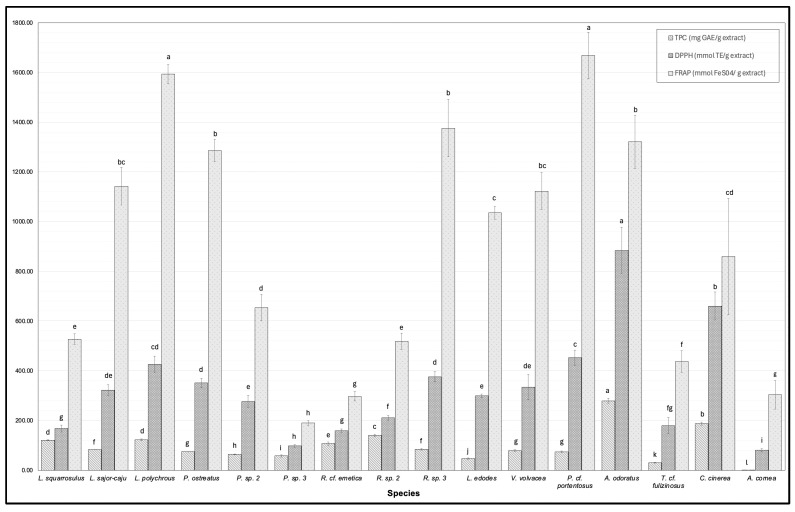
Total phenolic compounds (TPCs), DPPH radical scavenging activity, and ferric reducing antioxidant power assay of wild edible mushroom extracts (WEMs). Error bars represent standard deviations (*n* = 3). Significant differences among species in the same measurement are denoted by superscript letters (a–l) (*p* < 0.05).

The concentration of total phenolic compounds (TPCs), DPPH scavenging activity, and FRAP activity are presented in Figure 1. Phenolic compounds display diverse biological effects, for instance, antioxidant activities, antibacterial, anti-inflammatory, and antihyperglycemic effects [14]. TPCs exhibited significant variation across the WEM extracts, ranging from 0.88 ± 0.02 mg GAE/g for *A. cornea* to 278.13 ± 11.17 mg GAE/g for *A. odoratus*. Contrary findings have been reported in that the TPCs in *A. cornea* ranged from 8.40 to 2.78 mg GAE/g DW, varying with cooking methods [15], whereas another report observed TPCs ranging from 8.76 to 20.10 mg GAE/g DW in the fruiting body, depending on the species [12]. The differences observed in our study may be attributed to the hot water extraction method, which can influence the extraction efficiency of polyphenolic compounds from WEMs depending on their unique internal structures. In addition, variations in fungal growth conditions and nutrient availability could also have contributed to the observed differences.

The antioxidant activity was determined by employing the DPPH and FRAP assays and is depicted in Figure 1. The DPPH assay relies on the reactivity of DPPH with proton donors. As a stable free radical, DPPH reacts with antioxidants, serving as a measure of their capability to scavenge free radicals effectively. Figure 1 shows the DPPH scavenging activity of WEM extracts. Among the WEM extracts, DPPH was significantly varied, ranging from 80.33 ± 6.26 mmol TE/g for *A. cornea* to 884.08 ± 93.22 mmol TE/g for *A. odoratus*. The scavenging activity of the extracts followed the sequence *A. odoratus* > *C. cinerea* > *P.* cf. *portentosus* > *L. polychrous* > *R.* sp. 3 > *P. ostreatus* > *V. volvacea* > *L. sajor-caju* > *L. edodes* > *P.* sp. 2 > *R.* sp. 2 > *T.* cf. *fulizinosus* > *L. squarrosulus* > *R.* cf. *emetica* > *P.* sp. 3 > *A. cornea*. Notably, the DPPH scavenging activity exhibited a correlation with TPCs. Total phenols are widely recognized as natural antioxidants and significant contributors to the biological activities observed in medicinal plants, including mushrooms [46]. Our investigation is consistent with previous studies, revealing the strong positive correlation between TPCs and DPPH scavenging activity (r = 0.9851, R^2^ = 0.9705) [46]. It has been reported that gallic acid, protocatechuic acid, and catechin were prominently detected as the primary compounds across all wild edible mushrooms in Thailand [47]. Moreover, the effectiveness of the DPPH scavenging activity varies among individual phenolic compounds due to the presence of hydroxyl groups on their side chain functional groups ([18] I). Kaewnarin, Suwannarach [47] identified phenolic compounds in hot water extracts from wild edible mushrooms belonging to the Russulaceae family. *R.* cf. *emetica* contained gallic acid, protocatechuic acid, catechin, vanillic acid, rutin, and rosmarinic acid, while *R.* sp. contained gallic acid, protocatechuic acid, and catechin. Interestingly, the concentrations of gallic acid and catechin in *R.* sp. were approximately 3 and 2.5 times higher, respectively, compared to *R.* cf. *emetica*; additionally, *R.* sp. also showed markedly greater DPPH scavenging activity, which can be related to the enhanced antioxidant activities of gallic acid and catechin [47]. Gallic acid and catechin have been shown to possess exceptional antioxidant activity compared to other phenolic compounds [48]. This finding corresponds with our result where *R.* sp. had superior DPPH scavenging activity in comparison to *R.* cf. *emetica*.

Furthermore, the FRAP assay was employed to determine the ability of the antioxidant compound to convert ferric ions (Fe^3+^) to ferrous ions (Fe^2+^) through electron transfer. An increase in absorbance indicated higher antioxidant activity. The reductive capabilities of WEM extracts exhibited substantial variation, ranging from 190.26 ± 9.80 mmol FeSO_4_/g for *P.* sp. 3 to 1669.04 ± 93.57 mmol FeSO_4_/g for *P.* cf. *portentosus*. The FRAP activity of the extracts followed the sequence *P.* cf. *portentosus* > *R.* sp. 3 > *L. polychrous* > *A. odoratus* > *P. ostreatus* > *L. sajor-caju* > *V. volvacea* > *L. edodes* > *C. cinerea* > *P.* sp. 2 > *L. squarrosulus* > *R.* sp. 2 > *T.* cf. *fulizinosus* > *A. cornea* > *R.* cf. *emetica* > *P.* sp. 3. Interestingly, FRAP activity was slightly correlated to TPCs in a concentration-dependent manner. This aligns with a previous work that reported a strong association (r = 0.929) between the reducing power of mushroom samples and their phenolic levels. To summarize, our investigation results demonstrate a link between antioxidant activities and total phenolics, which agrees well with previous studies. This suggests that phenolics are the main component responsible for the antioxidant activity seen in mushrooms.

### 3.4. Phenolic Profile

The phenolic composition of the WEM extracts was analyzed using LC-MS, and the results are summarized in Table 3. Eight phenolic compounds were identified: P-coumaric acid, O-coumaric acid, quercetin, rutin, ethyl gallate, 3,4-dihydroxyphenylacetic acid (DOPAC), protocatechuic acid, and total catechin. Remarkably, P-coumaric acid, O-coumaric acid, quercetin, and rutin were commonly observed in most WEMs, with concentrations ranging from 0.04 ± 0.00 to 0.95 ± 0.00 mg/L, 0.31 ± 0.01 to 0.35 ± 0.05 mg/L, 1.57 ± 0.20 to 1.89 ± 0.00 mg/L, and 0.51 ± 0.00 to 0.53 ± 0.00 mg/L, respectively. Nonetheless, rutin was not detected in *L. squarrosulus*, *R.* cf. *emetica*, and *R.* sp. 3. Ethyl gallate, DOPAC, and protocatechuic acid were found in only a few WEMs. Interestingly, *A. odoratus* showed the highest concentrations of certain phenolic compounds among WEMs, including P-coumaric acid (0.95 ± 0.00 mg/L), ethyl gallate (11.17 ± 0.01 mg/L), and protocatechuic acid (90.52 ± 0.02 mg/L). These findings are consistent with the results of the TPC and antioxidant activity assays. Notably, protocatechuic acid exhibited a slight correlation with FRAP activity in a concentration-dependent manner, particularly in *A. odoratus* and *R.* sp. 3. Protocatechuic acid has been demonstrated to possess effective antioxidant activity by donating hydrogen atoms (H+) or electrons (e). Its structure includes –OH and –COOH functional groups, which enable it to chelate metal ions, such as ferrous ions. Generally, this chelating activity is attributed to the presence of ortho-dihydroxyl groups. Thereby, protocatechuic acid possibly emerges as a main phenolic compound in *A. odoratus*, serving as an antioxidant. Previous studies have demonstrated that protocatechuic acid possesses various pharmacological effects, serving as both an in vitro and in vivo antioxidant [49].

### 3.5. Important Antioxidant and Anti-Inflammatory Regulators

Nuclear factor erythroid 2-related factor 2 (Nrf2) is a key transcription factor that governs cellular responses to oxidative stress. It regulates the expression of numerous antioxidant and phase II enzyme genes [50]. Nrf2 also plays an essential role in modulating the anti-inflammatory response by controlling redox balance and activating antioxidant response element (ARE)-mediated anti-inflammatory genes. This includes the expression of Nrf2-regulated stress response enzymes, such as quinone oxidoreductase 1 (NQO1), a detoxification enzyme known for its anti-inflammatory effects. Activation of NQO1 contributes to the efficient detoxification of xenobiotics and enhanced antioxidant defense [50].

**Figure 2:**
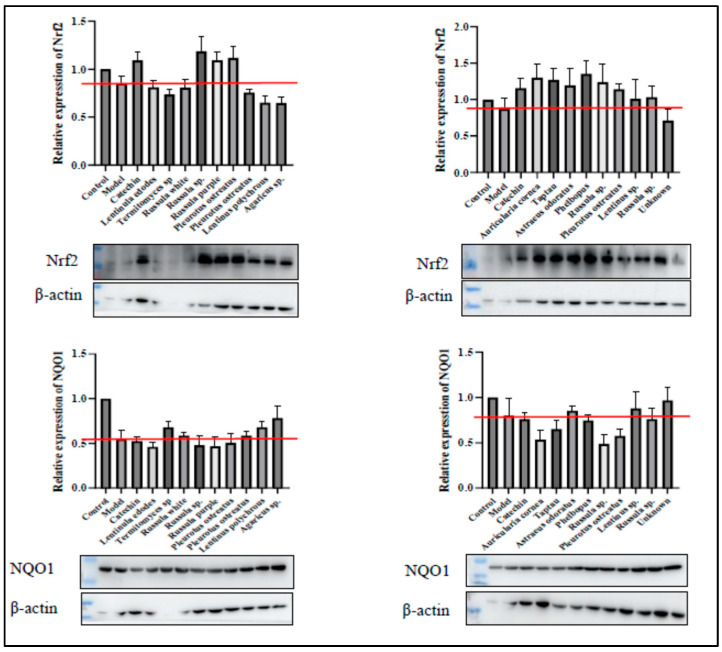
Effects of wild edible mushroom (WEM) extracts on the key antioxidant protein regulators. The proteins were detected by western blot analysis. The fold change in the protein concentration was calculated as the intensity of the treatment group relative to that of the control group and was normalized to that of β-actin by densitometry. The red line represents the protein expression level in the control (model) sample. *p* < 0.05.

This study aimed to evaluate the antioxidant and anti-inflammatory activities of WEM extracts and assess their potential as an Nrf2 activator. We also measured the levels of NQO1, a key phase II enzyme regulated by Nrf2. The relative expression of Nrf2 and NQO1, along with their protein profiles from SDS-PAGE western blot analysis are shown in Figure 2. Our findings suggest that the antioxidant and anti-inflammatory effects of WEM extracts are mediated through the activation of Nrf2-dependent antioxidant defense pathways. The protein expression levels for most WEM extracts displayed band densities comparable to that of catechin. Specifically, the relative expression levels of Nrf2 in *Russula* sp., *Russula purple*, *Pleurotus ostreatus*, *Auricularia cornea*, and *Auricularia odoratus* were greater than 1, showing similarities to catechin levels. The activation of Nrf2 is a powerful strategy for protecting against diseases related to oxidative stress [51]. Additionally, the NQO1 protein expression across all WEM extracts was similar to that of catechin, with comparable relative expression levels of NQO1 found in *Lebtinus* sp. Several medicinal mushrooms, such as *Antrodia salmonea* [52], *Ganoderma lucidum* [53], and *Mycoleptodonoides aitchisonii* [51], have been studied for their effects on the Nrf2 pathway and have shown the ability to prevent oxidative stress-related diseases by inducing antioxidant and phase II enzyme activities.

### 3.6. Digestive Enzyme Inhibition

**Figure 3:**
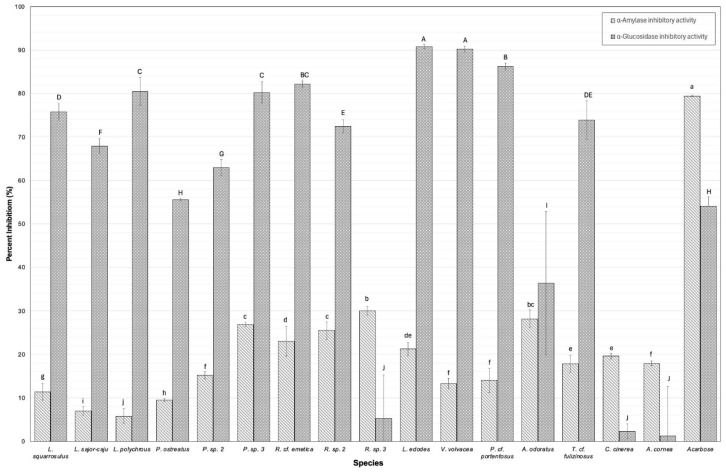
Inhibition of digestive enzymes by sixteen wild edible mushrooms (WEMs). Enzyme inhibition activity is expressed as a percentage (%) at a concentration of 0.063 mg/mL. Positive control, acarbose, was considered as 100% inhibition. Error bars represent the standard deviation (*n* = 3). Significant differences in enzyme inhibition activity among species are indicated by different lowercase letters (a–j) for the α-amylase inhibition and capital letters (A–H) for α-glucosidase inhibition (*p* < 0.05).

Digestive enzyme inhibition was investigated through the inhibitory activity against α-amylase and α-glucosidase enzymes in the lyophilized extract compared to acarbose, a drug used in controlling digestive enzymes in the small intestine for diabetes patients, and is presented in Figure 3. By slowing down the inhibition activity of these digestive enzymes, which indicate the digestion and absorption of carbohydrates, we can prevent the rapid rise in blood sugar levels following meals. This can be beneficial for managing type II diabetes and obesity [39]. The α-amylase and α-glucosidase inhibitory activities of each lyophilized extract at a concentration of 0.063 mg/mL are displayed in Figure 3. The results showed that the α-amylase inhibitory activity in WEM extracts varied significantly among species. The inhibitory activity (%) was observed, with values ranging from 5.82 ± 1.67 for *L. polychrous* to 30.03 ± 1.03 for *R.* sp. 3. In comparison, the positive control (acarbose) showed an inhibitory activity of 79.43 ± 0.23. The significantly highest values were observed to be similar between *R.* sp. 3 and *A. odoratus*. These findings correlate strongly with TPCs and antioxidant activities observed in *A. odoratus*. It exhibits notably high concentrations of TPCs along with antioxidant activities. Phenolic compounds, primarily flavonoids such as kaempferol and quercetin derivatives, are recognized for their capacity to inhibit α-amylase activity. This inhibition is crucial as it effectively delays the digestion and absorption of carbohydrates [39]. Notably, within the *Russulaceae* family, high inhibition against α-amylase activity was observed, ranging from 23.03 ± 3.38 to 30.03 ± 1.03%.

**Figure 4:**
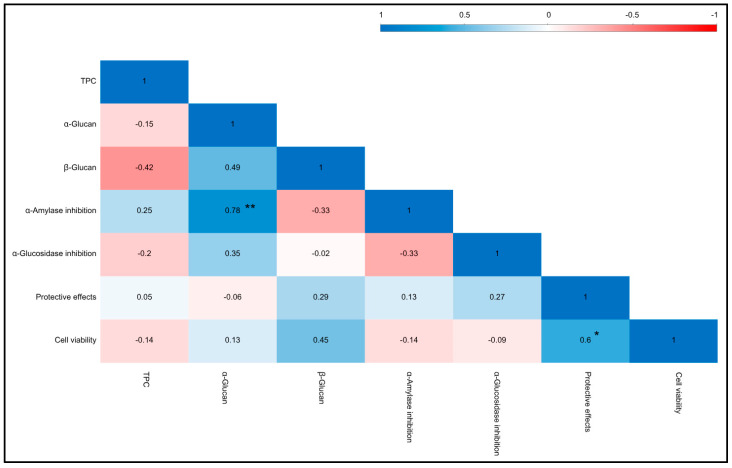
Pearson’s correlation coefficient among total phenolic compounds (TPCs), α-glucan, β-glucan, and bioactivities across mushroom species. (*) Correlation is significant at the 0.05 level (2-tailed). (**) Correlation is significant at the 0.01 level (2-tailed).

Li, Zhong [54] conducted a study demonstrating significant inhibition effects of water-soluble polysaccharides obtained from *R. virescens* against α-amylase, potentially attributable to the structural configuration and molecular weight distribution. In addition, the inhibitory activity against α-glucosidase (%) was observed, with values ranging from 2.31 ± 1.65 for *C. cinerea* to 90.79 ± 0.50 for *L. edodes*. In comparison, the positive control acarbose showed an inhibitory activity of 54.07 ± 2.12. The significantly highest values were observed to be similar in *L. edodes* and *V*. *volvacea*. Interestingly, *V. volvacea* contained a high concentration of glucans (i.e., β-glucan, α-glucan) among WEMs. Consequently, the concentration of glucan is a plausible determinant of the inhibitory activity against α-glucosidase. This might be due to the fact that the hydroxyl group on the β-glucan chain can form strong hydrogen bonds with α-glucosidase residues, thereby exerting inhibitory properties on the enzyme activity. Previous studies have also emphasized the potential of cereal β-glucan to reduce carbohydrate digestion, potentially lowering the risk of hyperglycemia and type II diabetes. Our investigation indicates that *R.* sp. 3, *A. odoratus*, *L. edodes*, and *V. volvacea* have the potential to function as inhibitors of α-glucosidase and α-amylase, thereby broadening their efficacy in the food industry. However, Pearson’s correlation coefficient revealed a statistically significant positive correlation (*p* < 0.01) between the α-glucan content and α-amylase inhibitory activity in Figure 4. This revealed that WEM extracts are inherently complex, comprising a diverse array of phenolics, proteins, polysaccharides, and other bioactive constituents. These compounds often coexist and may exert synergistic or antagonistic effects, complicating the attribution of the observed bioactivity to a single class of compounds without detailed fractionation and characterization [55]. For instance, phenolic compounds, including flavonoids and tannins, are well-known for their capacity to inhibit various enzymes differently. Proteins and peptides may also contribute to bioactivity through direct enzyme inhibition or by functioning as carriers that enhance the effectiveness of other molecules. Polysaccharides, especially those with sulfated groups, can indirectly influence enzyme activity by modulating the assay environment or exerting antioxidant effects.

### 3.7. Cytotoxicity and Protective Properties of WEMs on HepG2 Cells

Hepatoblastoma (HepG2) cells mimic liver tissue due to their roles in metabolizing nutrients from edible mushrooms into glucose, amino acids, and fatty acids. These cells also contain crucial enzymes necessary for essential metabolic functions [26]. HepG2 cell lines were exposed to varying concentrations (0.2 to 1 mg/mL) of WEMs. The potential cytotoxic effects of these mushroom extracts were evaluated using the CCK-8 assay. Table 4 illustrates the percentage of cell viability (%) at different concentrations of WEMs and their protective effects against tert-butyl hydroperoxide (tBPH). The findings showed that samples treated with WEM extracts maintained high cell viability compared to the positive control (catechin, 100), suggesting non-toxicity to the cells. For instance, incubating cells with the *A. odoratus* extract alone slightly decreased viability in a dose-dependent manner. At concentrations of 0.02, 0.1, 0.5, and 1 mg/mL, the extract reduced cell viability to 96.98 ± 13.06%, 94.61 ± 9.86%, 90.51 ± 11.55%, and 86.64 ± 8.39%, respectively. Indeed, these reductions are considerably not/less toxic to HepG2 cells, with cell viability consistently above 50%, a threshold often considered as the IC_50_.

Similarly, the majority of WEM extracts maintained high cell viability, suggesting they are safe for consumption. Furthermore, the protective effects against tBPH as indicated by % cell viability after exposure to tBPH were investigated. The results indicated that *R.* cf. *emetica* had the most efficacy in combating hepatotoxicity, with a significant viability rate of 86.51 ± 5.96% at a concentration of 0.02 mg/mL. In comparison, the positive control catechin showed inhibitory activity ranging from 88.20 ± 3.35 at a concentration of 10 µmol/L. It should be noted that the bioactivity measured through in vitro models does not fully reflect the complex physiological conditions of the human body. Further studies using in vivo models and human clinical trials are necessary to confirm the actual efficacy of the mushroom samples.

## 4. Conclusions

Significant variations in chemical compositions were observed among species, with subtle differences noted even within the same family. This study revealed a clear correlation between polyphenol concentrations, antioxidant activities, and key antioxidant regulators, with *A. odoratus* demonstrating particularly potent antioxidant and anti-inflammatory properties. Both *R.* sp. 3 and *A. odoratus* exhibited the highest α-amylase inhibitory activity, while *L. edodes* and *V. volvacea* showed the strongest α-glucosidase inhibitory effects. These activities were attributed to the presence of glucans (such as β-glucan and α-glucan) and polyphenolic compounds, indicating a potential delay in carbohydrate digestion. Cell viability remained consistently high (>80%), indicating safety at the studied concentrations, while hepatotoxicity in HepG2 cells was reduced by over 50%, suggesting a protective effect against liver toxicity. These findings highlight that WEMs are not only rich sources of nutritional and bioactive compounds but also promising candidates for the food industry, particularly functional ingredients and non-animal-based meat production. Furthermore, the rising demand for sustainable protein sources has driven the large-scale cultivation and commercialization of wild edible mushrooms, further enhancing their potential in this sector. These findings underscore that wild edible mushrooms are rich in both nutritional and bioactive compounds, positioning them as promising candidates for the food industry, particularly in non-animal-based meat production. Furthermore, the growing demand for sustainable protein sources has fueled the expansion of wild edible mushroom cultivation and commercialization, significantly enhancing their potential in the market. WEMs have the potential for international recognition, supported by scientific data, contributing to the economic development of local communities, promoting sustainable culinary practices, and preserving cultural heritage.

## Figures and Tables

**Table 1:** Morphological and molecular identification of sixteen wild edible mushrooms.

No.	Family	Species	Thai Common Name	DNA Sequence	Number of Bases
**1**	Polyporaceae	*Lentinus squarrosulus*	Hed lom	CTTTGGGTATGTGCACATCCTCCTCCGATTTCTATTCATCCACCTGTGCACTTTTTGTAGAGTTCTTTCATCGGGTTTTTGAAGGTGCTCATTATGAGTTACTTGAAAAGACTAGTTGACAAGGCTTCTATGTTCTTATAAACCATTGAAGTATGTTATAGAATGATCTTGTTATTGGGACTTTATTGACCCTTTAAACTTAATACAACTTTCAGCAACGGATCTCTTGGCTCTCCCATCGATGAAGAACGCAGCGAAATGCGATAAGTAATGTGAATTGCAGAATTCAGTGAATCATCGAATCTTTGAACGCACCTTGCGCCCTCTGGTATTCCGGAGGGCATGCCTGTTTGAGTGTCATTAAATTCTCAACTTTATAAGTTTTT	387
**2**	Polyporaceae	*Lentinus sajor-caju*	Hed lom	GAAGTAAAAGTCGTAACAAGGTTTCCGTAGGTGAACCTGCGGAAGGATCATTATCGAGTTATTGAAACGGGTTGTAGCTGGCCTTACGAGGCATGTGCACGCCCTGCTCATCCACTCTACACCTGTGCACTTACTGTGGGTTTCAGGAGCTTCGAAAGCGGAGGGCCTTTGCGGGCTTTTCGTTATTAGTTGTGACTGGGCTCATGTCCACTACACACTCTTATAAAGTAACAGAATGTGTATTGCGATGTAACGCATCTATATACAACTTTCAGCAACGGATCTCTTGGCTCTCGCATCGATGAAGAACGCAGCGAAATGCGATAAGTAATGTGAATTGCAGAATTCAGTGAATCATCGAATCTTTGAACGCACCTTGCGCTCCTTGGTATTCCGAGGAGCATGCCTGTTTGAGTGTCATGAAATTCTCAACCTGACGGGTTCTTAACGGAGCTTGGTTCAGGCTTGGACTTGGAGGCTTGTCGGCTTGCTTTGTCGAGTCGGCTCCTCTCAAATGCATTAGCTTGGTTCTTTGCGGATCGGCTCACGGTGTGATAATTGTCTACGCCGCGACCGTTGAAGCGTTTGAATGGGCCAGCTTATAGTCGTCTCCATCGCGAGACAACATTTCATCGAACTCTGACCTCAAATCAGGTAGGACTACCCGCTGAACTTAAGCATATCATAA	445
**3**	Polyporaceae	*Lentinus polychrous*	Hed lom	CCTGCGGAAGGATCATTATCGAGTCTTGAAACGGGTTGTAGCTGGCCTTCCGAGGCATGTGCACGCCCTGCTCATCCACTCTACACCTGTGCACTTACTGTGGGTTTCAGGAGCTTCGAAAGCGAGAAGGGCCCCTYGGGGGGTCAGTCTCGTCGTAGTAGTGACTGGGCCCACGTTTACTATAAACTCTTAAAAGTATCAGAATGTGTATTGCGATGTAACGCATCTATATACAACTTTCAGCAACGGATCTCTTGGCTCTCGCATCGATGAAGAACGCAGCGAAATGCGATAAGTAATGTGAATTGCAGAATTCAGTGAATCATCGAATCTTTGAACGCACCTTGCGCTCCTTGGTATTCCGAGGAGCATGCCTGTTTGAGTGTCATGAAATTCTCAACCTAGCGGGTTCTTAACCGGACTTGCTTAGGCTTGGACTTGGAGGCTTTGTCGGCTTGCTTATGTCGAGTCGGCTCCTCTCAAATGCATTAGCTTGGTTCCTTTGCGGATCGGCTCACGGTGTGATAATTGTCTACGCCGCGACCGTTGAAGCGTTTTAATGGCCAGCTTCTAATCGTCTCTTGCGAGACAACATTCATCGAACTCCTGACC	612
**4**	Pleurotaceae	*Pleurotus ostreatus*	Hed nang fah	Identified by morphology	
**5**	Pleurotaceae	*Pleurotus* sp. 2	Hed nang fah	Identified by morphology	
**6**	Pleurotaceae	*Pleurotus* sp. 3	Hed nang fah	Identified by morphology	
**7**	Russulaceae	*Russula* cf. *emetica*	Hed lom krakeaw	Identified by morphology	
**8**	Russulaceae	*Russula* sp. 2	Hed deang nhammak	GGGCTGTCGCTGACCTTTGCGGGTCGTGCACGCTCAAAGTGCTCTCTCTCATATCCAACTCACCCCTTTGTGCATCACCGCGTGGGCCCCACCCTCTTGGGATCGGTTCACGTTTTTCTACAAACACCTTTCTTTTAATGCATGTGTAGAATGTCTTCCTTTTTTGCGATCACGCGCAATCAATACAACTTTCAACAACGGATCTCTTGGCTCTCGCATCGATGAARAACGCAGCGAAATGCGATACGTAATGTGAATTGCAGAATTCAGTGAATCATCGAATCTTTGAACGCACCTTGCGCCCCTTGGTATTCCGAGGGGCACACCCGTTTGAGTGTCGTGAACATCCTCAACCTTTTTGGTTTCTTGACCAAGAAGGCTTGGACTTTGGAGGTTTATCCTTGCTGGCTTACCTTTGAAGCCAGCTCCTCCTAAATGAATTAKTGGGGTCCCCTTTGCCGATCCTTGATGTGATAAGTATTGCTTCTACGTCTTGGGTCTCGCACTGTTGCCCTGGAACCCCSCYTYCMACMRKCYTTCTTTYCAAAAASAATGTYGAAKTGGMTTGGTACTCCAMTCTCTACCAACTTGAACTCCAAWYSGGKGARAATAACCGCTGGAMTTWAKCATAATCAAA	637
**9**	Russulaceae	*Russula* sp. 3	Hed nha meung	GCTGTCGCTGACCTTTGCGGGTCGTGCACGCTCAAAGTGCTCTCTCTCATATCCAACTCACCCCTTTGTGCATCACCGCGTGGGCCCCACCCTCTTGGGATCGGTTCACGTTTTTCTACAAACACCTTTCTTTTAATGCATGTGTAGAATGTCTTCCTTTTTTGCGATCACGCGCAATCAATACAACTTTCAACAACGGATCTCTTGGCTCTCGCATCGATGAARAACGCAGCGAAATGCGATACGTAATGTGAATTGCAGAATTCAGTGAATCATCGAATCTTTGAACGCACCTTGCGCCCCTTGGTATTCCGAGGGGCACACCCGTTTGAGTGTCGTGAACATCCTCAACCTTTTTGGTTTCTTGACCAAGAAGGCTTGGACTTTGGAGGTTTATCCTTGCTGGCTTACCTTTGAAGCCAGCTCCTCCTAAATGAATTAKTGGGGTCCCCTTTGCCGATCCTTGATGTGATAAGTATTGCTTCTACGTCTTGGGTCTCGCACTGTTGCCCTGGAACCCCSCYTYCMACMRKCYTTCTTTYCAAAAASAATGTYGAAKTGGMTTGGTACTCCAMTCTCTACCAACTTGAACTCCAA	597
**10**	Marasmiaceae	*Lentinula edodes*		ATTGTTGCTGGCCTTTGGGTATGTGCACATCCTCCTCCGATTTCTATTCATCCACCTGTGCACTTTTTGTAGGAGTTCTTTCATCGGGTTTTTGAAGGTGCTCATTATGAGTTACTTGAAAAGACTAGTTGACAAGGCTTCTATGTTCTTATAAACCATTGAAGTATGTTATAGAATGATCTTGTTATTGGGACTTTATTGACCCTTTAAACTTAATACAACTTTCAGCAACGGATCTCTTGGCTCTCCCATCGATGAAGAACGCAGCGAAATGCGATAAGTAATGTGAATTGCAGAATTCAGTGAATCATCGAATCTTTGAACGCACCTTGCGCCCTCTGGTATTCCGGAGGGCATGCCTGTTTGAGTGTCATTAAATTCTCAACTTTATAAGTTTTTAMTTATTAAARSTTGGAAGGTGGAAGSTTGGMRGGSKTTGGCMRCTCCTCCTAAATTTATTAAKGGGAACCCTGGTTTGGTARTTCYAACCTTGGKGGGAAAATTATCTAC	510
**11**	Pluteaceae	*Volvariella volvacea*	Hed fang	TGCTGGCTCCTCGGAGCAGGTGCACGCCCTCCCCGACGCCTTCCATTCTCCACGTCCCCACCTGTGCACCTTCTGTAGGCCGTGAAGCCGCCTCGTTCGGCTCCCTCGGCTCTACGAGATCTTTTGTACACCCTTGAGAAAAACGTGTTGCAGAGTGTTCTTGTACGACCGGGGACCCCTCGTCGGCCCCATAGACATACCAATACAACTTTCAACAACGGATCTCTTGGCTCTCGCATCGATGAAGAACGCAGCGAAATGCGATAAGTAATGTGAATTGCAGAATTCAGTGAATCATCGAATCTTTGAACGCACCTTGCGCTCTTTGGCCATTCCGAAGAGCATGCCTGTTTGAGTGTCATCGAATCCTCAAGCCCAGCCCGGCTTCTCCCCGGGCTTTTGGGGGCTTGGAGTTGGGAGCTGTGCGGGTCGCTAGCCTTCGCGATCCGCTCTCCTCAAAGGCATCAGCAGGGCCCAGTCGCAGTCGGCCTCGTGGCGTTGATAGTCCATCTACGCCCCCCCGCGGCCGCACTCAGCGTGGCTCGGCTTCGAACCGTCCGGAC	563
**12**	Boletinellaceae	*Phlebopus* cf. *portentosus*		Identified by morphology	
**13**	Diplocystaceae	*Astraeus odoratus*		AAAGATTTCCGGCCCCGGGATTAACAACCCCGGCGCGGCGCGGCAACATGGTTGGAAGCATGAGACACGTCGATCCAAGCACTTCCAGCCCACGACGATCACTACGACGTCGAACAGGCCGTGCCGTGCAAAGGCTCGAAGCCCACCGCTAATGCATTTGAGGAGAGCCGGCGTCCCGAGCAAAGTCGGGTCGCCCGCAGACTCCCAAAGTCCAAGTCCGAGCTCGCTCCGAGTCGGCGACCGAAGCAAAGCTTAGGATTTGAGATTTCGATGACACTCAAACAGGCATGCTCCTCGGAATACCAAGGAGCGCAAGGTGCGTTCAAAGATTCGATGATTCACGGAAAATCTGCAATTCACATTACTTATCGCGATTCGCTGCGTTCTTCATCGATGCGAGAGCCAAGAGATCCATTGCTGAAAGTTATATATATGTTTATATGACATGTTTGTCAAAAGACAACGTTCTGTATACATGCAGAGAGCTTTATAAAAA	496
**14**	Lyophyllaceae	*Termitomyces* cf. *fulizinosus*	Hed kone	Identified by morphology	
**15**	Psathyrellaceae	*Coprinopsis cinerea*	Hed hu noo	Identified by morphology	
**16**	*Auriculariaceae*	*Auricularia cornea*		CTTGGTCATTTAGAGGAAGTAAAAGTCGTAACAAGGTTTCCGTAGGTGAACCTGCGGAAGGATCATTAAAGATTTTGGGCTTTTAacCCGATCGTTCAGCTGTGCGCCCTTCACAGGGCTGCACGCTGGAGCAAGACCCCACACCTGTGCACCTTTTCGGTTGCGGCTTCGGTCGCTGCCGCTTTCAAATGCAACAACTCAGTCTCGAATGTTAACAAAACCATAAAAAGTAACAACTTTCAACAACGGATCTCTTGGCTCTCGCATCGATGAAGAACGCAGCGAAATGCGATAAGTAATGTGAATTGCAGAATTCAGTGAATCATCGAATCTTTGAACGCATCTTGCGCTCCTTGGTATTCCATGGAGCATGCCTGTTTGAGTGTCACGTAAACCCTCACCCTTGCGATGTAACAGTCGCCCGTGGTGGACTTGGACTGTGCCGTAACCGGCTCGTCTTGAAATGCATTAGCTGGCGCTTTTAGAGTGCTGGGCGACGGTGTGATAATTATCTGCGCCAATGCCTTAGGCCTCTTCAGCGGTGCTGCTTACAGCCGTCCCTCTGTGGACACATTATTTTTAAAGCTTTGGCCTCAAATCAGGTAGGACTACCCGCTGAACTTAAGCATATCAATAAGCGGAGGAA	646

**Table 2:** Macronutrients and functional polysaccharides of sixteen wild edible mushrooms.

Species	Crude Fat ^A^	Ash ^A^	Crude Fiber ^A^	Protein ^A^	β-Glucan ^B^	α-Glucan ^B^	Total Glucan ^B^
***L. squarrosulus***	1.16 ± 0.04 ^c^	8.56 ± 0.03 ^abc^	21.26 ± 0.86 ^f^	31.96 ± 0.54 ^bc^	5.00 ± 0.97 ^cd^	2.41 ± 0.09 ^cdef^	7.41 ± 0.99 ^efgh^
***L. sajor-caju***	1.31 ± 0.05 ^c^	5.41 ± 0.85 ^def^	23.42 ± 0.58 ^e^	19.84 ± 0.38 ^f^	16.69 ± 0.89 ^b^	8.40 ± 1.36 ^a^	25.08 ± 0.99 ^b^
***L. polychrous***	0.83 ± 0.12 ^c^	5.41 ± 0.11 ^def^	30.08 ± 1.34 ^b^	28.42 ± 0.34 ^c^	5.06 ± 1.42 ^cd^	6.34 ± 0.44 ^b^	11.40 ± 0.99 ^de^
***P. ostreatus***	1.12 ± 0.05 ^c^	5.81 ± 0.09 ^cde^	23.68 ± 0.94 ^d^	22.10 ± 0.17 ^e^	15.64 ± 2.12 ^b^	7.74 ± 1.35 ^a^	23.37 ± 0.99 ^b^
***P. *** **sp. 2**	1.04 ± 0.11 ^c^	4.66 ± 0.08 ^def^	18.49 ± 1.12 ^j^	26.74 ± 0.26 ^d^	5.23 ± 1.01 ^cd^	2.18 ± 0.07 ^def^	7.41 ± 0.99 ^efgh^
***P. *** **sp. 3**	0.91 ± 0.05 ^c^	3.25 ± 0.03 ^ef^	23.26 ± 2.29 ^e^	23.74 ± 0.26 ^e^	9.19 ± 0.94 ^c^	1.64 ± 0.06 ^defg^	10.83 ± 0.99d ^ef^
***R. *** **cf. ** ***emetica***	1.38 ± 0.06 ^c^	9.32 ± 0.26 ^ab^	19.29 ± 1.61 ^i^	31.74 ± 2.33 ^bc^	9.64 ± 4.55 ^c^	0.62 ± 0.05 ^g^	10.26 ± 4.52 ^defg^
***R. *** **sp. 2**	2.38 ± 0.29 ^b^	8.62 ± 0.10 ^abc^	17.87 ± 1.02 ^j^	30.15 ± 0.39 ^c^	6.41 ± 1.64 ^cd^	0.43 ± 0.09 ^g^	6.84 ± 1.71 ^fgh^
***R. *** **sp. 3**	4.71 ± 0.03 ^a^	9.21 ± 0.03 ^ab^	20.27 ± 1.21 ^h^	30.93 ± 0.23 ^c^	6.33 ± 1.14 ^cd^	1.53 ± 0.07 ^defg^	7.86 ± 1.20 ^efgh^
***L. edodes***	0.78 ± 0.04 ^c^	6.16 ± 0.12 ^cde^	17.65 ± 0.58 ^j^	21.99 ± 0.79 ^e^	9.45 ± 0.25 ^c^	3.66 ± 0.74 ^c^	13.11 ± 0.99 ^d^
***V. volvacea***	1.50 ± 0.12 ^b^	5.54 ± 0.40 ^cde^	20.53 ± 1.47 ^g^	30.85 ± 0.59 ^c^	23.86 ± 7.66 ^a^	6.35 ± 1.82 ^b^	30.21 ± 6.01 ^a^
***P. *** **cf. ** ***portentosus***	0.52 ± 0.01 ^d^	9.19 ± 0.07 ^ab^	11.75 ± 0.91 ^k^	33.89 ± 1.41 ^b^	7.94 ± 0.43 ^c^	5.74 ± 0.43 ^b^	13.68 ± 0.00 ^d^
***A. odoratus***	2.05 ± 0.16 ^b^	11.49 ± 1.13 ^a^	31.14 ± 3.67 ^a^	35.11 ± 2.82 ^b^	2.21 ± 1.00 ^d^	1.78 ± 0.07 ^defg^	3.99 ± 0.99 ^h^
***T. *** **cf. ** ***fulizinosus***	0.91 ± 0.05 ^c^	3.25 ± 0.03 ^ef^	0.91 ± 0.05 ^c^	23.74 ± 0.26 ^e^	4.64 ± 0.99 ^cd^	1.63 ± 0.06 ^defg^	6.27 ± 0.99 ^gh^
***C. cinerea***	1.34 ± 0.02 ^c^	7.41 ± 0.08 ^bcd^	1.34 ± 0.02 ^c^	51.87 ± 1.05 ^a^	9.30 ± 6.20 ^c^	1.53 ± 1.55 ^defg^	10.83 ± 5.50 ^def^
***A. cornea***	0.76 ± 0.01 ^c^	2.52 ± 0.04 ^f^	28.94 ± 1.35 ^c^	12.02 ± 0.26 ^g^	17.56 ± 2.09 ^b^	1.26 ± 1.25 ^efg^	18.81 ± 1.71 ^c^

Values are presented as mean ± SD (*n* = 3). Different letters (a–k) indicate significant differences in the same column (*p* < 0.05). ^A^ reported as g/100 g dry sample weight basis, ^B^ reported as g/100 g wet sample weight basis.

**Table 3:** Phenolic composition of sixteen wild edible mushrooms (mg/L).

Species	P-Coumaric Acid	O-Coumaric Acid	Quercetin	Rutin	Ethyl Gallate	DOPAC	Protocate Chuic Acid	Total Catechin
***L**. squarrosulus*	0.05 ± 0.02 ^b^	0.32 ± 0.02 ^a^	1.85 ± 0.03 ^a^	ND	ND	ND	4.80 ± 2.54 ^d^	2.87 ± 0.93 ^b^
***L**. sajor-caju*	0.05 ± 0.01 ^b^	0.32 ± 0.01 ^a^	1.88 ± 0.01 ^a^	0.51 ± 0.00 ^a^	ND	32.32 ± 0.05 ^d^	ND	1.78 ± 0.17 ^cd^
***L**. polychrous*	0.05 ± 0.00 ^b^	0.32 ± 0.01 ^a^	1.88 ± 0.00 ^a^	0.51 ± 0.00 ^a^	2.17 ± 0.00 ^c^	25.81 ± 0.00 ^e^	ND	1.85 ± 0.05 ^c^
***P**. ostreatus*	0.06 ± 0.00 ^b^	0.33 ± 0.01 ^a^	1.88 ± 0.00 ^a^	0.51 ± 0.00 ^a^	ND	ND	ND	1.64 ± 1.17 ^d^
***P**.* sp. 2	0.07 ± 0.01 ^b^	0.34 ± 0.01 ^a^	1.88 ± 0.00 ^a^	0.52 ± 0.01 ^a^	ND	ND	6.15 ± 0.03 ^d^	ND
***P**.* sp. 3	0.04 ± 0.00 ^b^	0.31 ± 0.02 ^a^	1.88 ± 0.00 ^a^	0.52 ± 0.03 ^a^	ND	ND	58.08 ± 0.05 ^c^	1.84 ± 0.02 ^c^
***R**.* cf. *emetica*	0.05 ± 0.01 ^b^	0.31 ± 0.00 ^a^	1.86 ± 0.01 ^a^	ND	ND	68.84 ± 0.15 ^a^	ND	1.56 ± 1.35 ^e^
***R**.* sp. 2	0.05 ± 0.01 ^b^	0.32 ± 0.00 ^a^	1.88 ± 0.01 ^a^	0.52 ± 0.01 ^a^	ND	64.97 ± 0.01 ^b^	77.42 ± 58.54 ^b^	0.39 ± 0.01 ^h^
***R**.* sp. 3	0.05 ± 0.00 ^b^	0.31 ± 0.01 ^a^	1.89 ± 0.00 ^a^	ND	9.80 ± 0.01 ^b^	ND	56.31 ± 24.88 ^bc^	0.80 ± 0.67 ^f^
***L**. edodes*	0.09 ± 0.35 ^ab^	0.31 ± 0.02 ^a^	1.57 ± 0.20 ^b^	0.53 ± 0.01 ^a^	ND	ND	ND	3.37 ± 0.13 ^ab^
***V**. volvacea*	0.09 ± 0.02 ^a^	0.35 ± 0.02 ^a^	1.83 ± 0.01 ^a^	0.52 ± 0.02 ^a^	ND	ND	ND	0.71 ± 0.14 ^g^
***P**.* cf. *portentosus*	ND	0.31 ± 0.01 ^a^	1.88 ± 0.01 ^a^	0.52 ± 0.01 ^a^	ND	ND	7.08 ± 0.01	2.39 ± 0.08 ^b^
***A**. odoratus*	0.95 ± 0.00 ^a^	0.32 ± 0.01 ^a^	1.88 ± 0.00 ^a^	0.51 ± 0.00 ^a^	11.17 ± 0.01 ^a^	ND	90.52 ± 0.02 ^a^	1.34 ± 0.90 ^ed^
***T**.* cf. *fulizinosus*	0.05 ± 0.01 ^b^	0.32 ± 0.00 ^a^	1.88 ± 0.01 ^a^	0.51 ± 0.00 ^a^	ND	ND	ND	0.41 ± 0.01 ^h^
***C**. cinerea*	0.07 ± 0.01 ^b^	0.34 ± 0.00 ^a^	1.88 ± 0.00 ^a^	0.51 ± 0.01 ^a^	ND	50.35 ± 0.02 ^c^	ND	ND
***A**. cornea*	0.05 ± 0.00 ^b^	0.32 ± 0.01 ^a^	1.88 ± 0.00 ^a^	0.51 ± 0.01 ^a^	ND	13.50 ± 14.67 ^f^	ND	3.81 ± 0.28 ^a^

Values are presented as mean ± SD (*n* = 3). ND: not detected, DOPAC: 3,4-Dihydroxyphenylacetic acid. Significant differences among species in the same phenolic compound are denoted by superscript letters (a–h) (*p* < 0.05).

**Table 4:** Cell viability (cytotoxicity) and protective effects on HepG2 cells against tBPH (hepatotoxicity) of sixteen wild edible mushrooms.

Species	Cell Viability (%)				Protective Effects on HepG2 Cells (mg/mL)
	Concentration of Mushroom Extract (mg/mL)				
	0.02	0.1	0.5	1	
***Model ^A^***	NA	NA	NA	NA	44.54 ± 6.20
***Positive ^B^***	NA	NA	NA	NA	88.20 ± 3.35
*L. sq**uarrosulus***	95.26 ± 5.54	94.73 ± 8.32	95.37 ± 4.46	92.28 ± 7.14	50.86 ± 3.37
*L. **sajor-caju***	100.63 ± 9.93	103.08 ± 8.93	96.44 ± 10.43	92.72 ± 5.43	59.04 ± 12.54
*L. **polychrous***	95.26 ± 5.54	94.73 ± 8.32	95.37 ± 4.46	92.28 ± 7.14	50.86 ± 3.37
*P.** ostreatus***	101.43 ± 3.70	103.07 ± 0.58	104.98 ± 10.27	104.06 ± 8.32	50.36 ± 5.12
***P. *** **sp. 2**	104.46 ± 6.23	108.91 ± 10.59	109.51 ± 14.21	107.55 ± 10.55	58.99 ± 3.29
***P. *** **sp. 3**	95.03 ± 9.48	102.46 ± 7.68	102.10 ± 12.48	93.19 ± 8.77	51.21 ± 2.60
*R. *c**f. *****emetica***	97.83 ± 5.47	97.90 ± 6.90	103.15 ± 8.15	104.01 ± 14.84	86.51 ± 5.96
***R. *** **sp. 2**	99.09 ± 6.57	93.72 ± 11.32	95.74 ± 9.74	94.33 ± 9.67	62.86 ± 11.14
***R. *** **sp. 3**	99.72 ± 4.38	100.67 ± 1.04	102.79 ± 4.69	100.58 ± 1.44	61.98 ± 10.56
***L. edodes***	97.69 ± 3.13	102.20 ± 14.25	94.79 ± 14.59	89.15 ± 19.88	52.84 ± 8.26
*V**. volvacea***	105.49 ± 6.13	103.73 ± 5.23	99.72 ± 3.99	107.54 ± 7.81	77.39 ± 8.15
*P. *cf. *p**ortentosus***	100.58 ± 3.26	104.95 ± 11.58	97.85 ± 9.93	98.35 ± 10.41	60.37 ± 6.36
*A**. odoratus***	96.98 ± 13.06	94.61 ± 9.86	90.51 ± 11.55	86.64 ± 8.39	50.36 ± 11.78
*T. *cf. *f**ulizinosus***	97.53 ± 8.89	97.16 ± 5.24	87.17 ± 13.13	87.20 ± 11.03	51.71 ± 11.43
***C. cinerea***	106.50 ± 11.23	108.37 ± 7.25	103.63 ± 9.03	102.23 ± 3.48	58.61 ± 6.55
***A. cornea***	98.31 ± 7.44	96.17 ± 6.17	96.35 ± 5.21	94.69 ± 5.29	46.41 ± 2.65

Values are presented as mean ± SD (*n* = 3). NA: not available. ^A^ treatment with tBPH without any extracts, ^B^ treatment with tBPH with catechin 10 µmol/L

## Data Availability

The original contributions presented in the study are included in the article/Supplementary Material, further inquiries can be directed to the corresponding author.

## Supplementary Materials

The following supporting information can be downloaded at: https://www.mdpi.com/article/10.3390/foods14132331/s1, Table S1. Limit of detection (LOD) and retention time of phenolic compounds analyzed in this study using LC-MS; Figure S1. The chromatogram of phenolic compounds analyzed in this study using LC-MS, found in wild edible mushroom.
