# Supplementary material for: Indigenous Wild Edible Mushrooms: Unveiling the Chemical Compositions and Health Impacts
Source: Foods. 2025 Jun 30;14(13):2331. doi: 10.3390/foods14132331 (PMC12249159; doi:10.3390/foods14132331)
Supplement: Supplementary file 1 [file foods-14-02331-s001.zip › foods-3687042-supplementary.pdf]

SUPPLEMENTAL FILES

Table S1. Limit of detection (LOD) and retention time of phenolic compounds analyzed in this study using LC-MS.

Phenolic compound	LOD (mg/L)	Retention time (min)
**O-Coumaric acid**	0.05	6.189
**P-Coumaric acid**	0.03	6.188
**Quercetin**	0.06	6.872
**Rutin**	0.03	6.347
**Ethyl gallate**	0.03	5.416
**3,4-Dihydroxyphenylacetic acid (DOPAC)**	0.06	3.902
**Protocatechuic acid**	4.42	2.725
**Total catechins**		
**Catechin gallate (CG)**	0.03	6.349
**Epigallocatechin gallate (EGCG)**	0.03	5.518
**Gallocatechin (GC)**	0.03	2.670

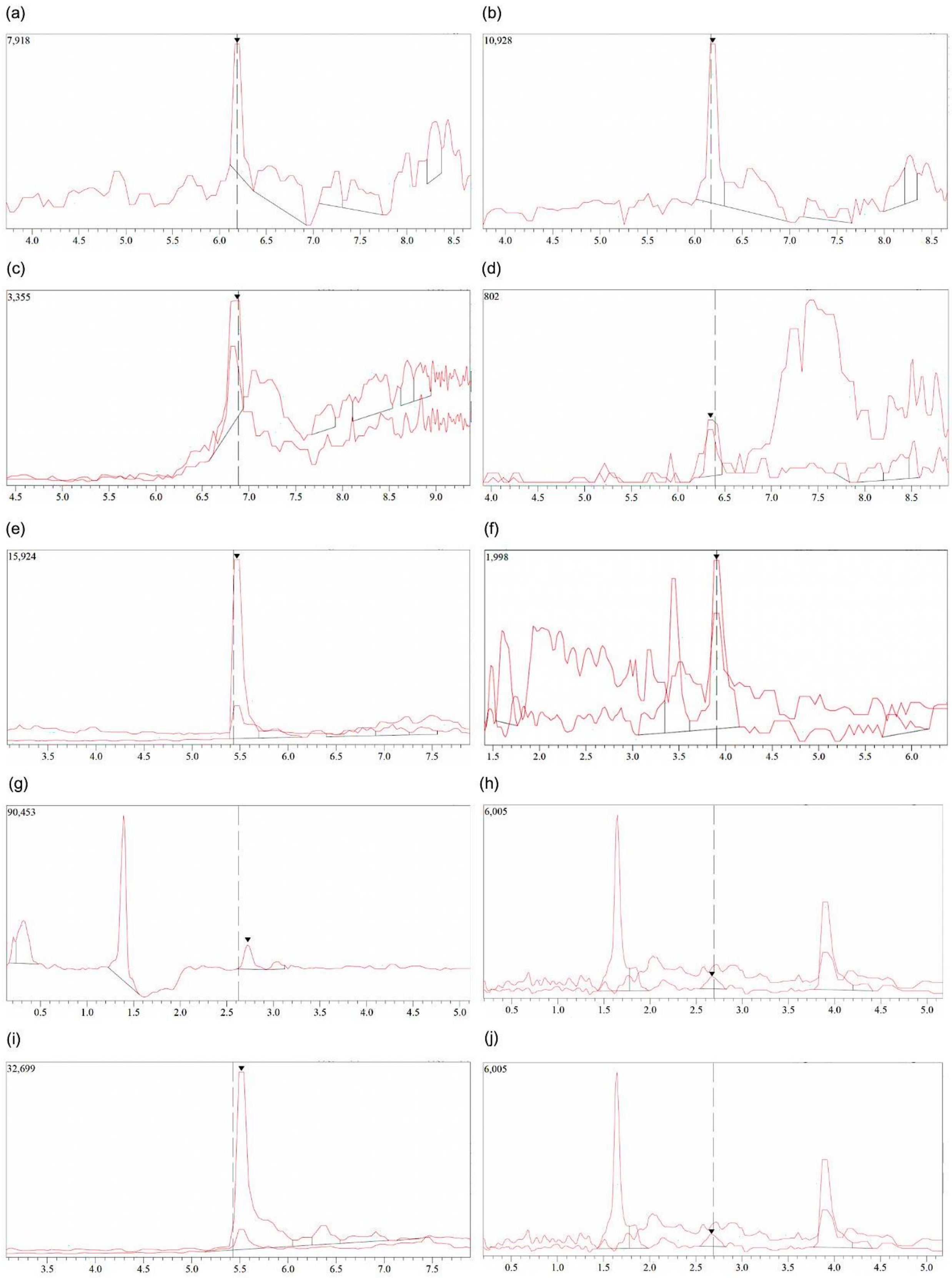
(a)
7,918
(b)
10,928
(c)
3,355
(d)
802
(e)
15,924
(f)
1,998
(g)
90,453
(h)
6,005
(i)
32,699
(j)
6,005

Figure S1. The chromatogram of phenolic compounds analyzed in this study using LC-MS, found in wild edible mushroom. (a): O-coumaric acid; (b): P-coumaric acid; (c): quercetin; (d): rutin; (e): ethyl gallate; (f): 3,4-dihydroxyphenylacetic acid; (g): protocatechuic acid; (h): catechin gallate; (i): epigallocatechin gallate; (j): gallocatechin.
